# Supplementary material for: Observation of Magnetic Radial Vortex Nucleation in a Multilayer Stack with Tunable Anisotropy
Source: Sci Rep. 2018 May 8;8:7180. doi: 10.1038/s41598-018-25392-x (PMC5940785; doi:10.1038/s41598-018-25392-x)
Supplement: Supplementary file 1 — Supplementary Information [file 41598_2018_25392_MOESM1_ESM.pdf]

Observation of Magnetic Radial Vortex Nucleation in a Multilayer Stack with Tunable Anisotropy

Vedat Karakas[1*], Aisha Gokce[1], Ali Taha Habiboglu[1], Sevdenur Arpaci[1], Kaan Ozbozduman[1], Ibrahim Cinar[1,2], Cenk Yanik[3], Riccardo Tomasello[4], Silvia Tacchi[5], Giulio Siracusano[6], Mario Carpentieri[7], Giovanni Finocchio[6], Thomas Hauet[8], Ozhan Ozatay[1]

[1]Physics Department, Bogazici University, Bebek 34342, Istanbul, TURKEY

[2]Karamanoglu Mehmetbey University, Department of Physics, 70100 Karaman, TURKEY

[3]Sabanci University Nanotechnology Research and Application Center Tuzla, 34956 Istanbul, TURKEY

[4]Department of Engineering, Polo Scientifico Didattico di Terni, University of Perugia, Terni, ITALY

[5]Istituto Officina dei Materiali del CNR (CNR-IOM), Sede Secondaria di Perugia, c/o Dipartimento di Fisica e Geologia, Università di Perugia, Perugia, ITALY

[6]Department of Mathematical and Computer Sciences, Physical Sciences and Earth Sciences, University of Messina, Messina, ITALY

[7]Department of Electrical and Information Engineering, Politecnico di Bari, I-70125 Bari, ITALY

[8]Institut Jean Lamour, UMR CNRS-Université de Lorraine, 54506 Vandoeuvrelès Nancy, FRANCE

Supplementary Information

Further Statistics for Radial Vortex Motion

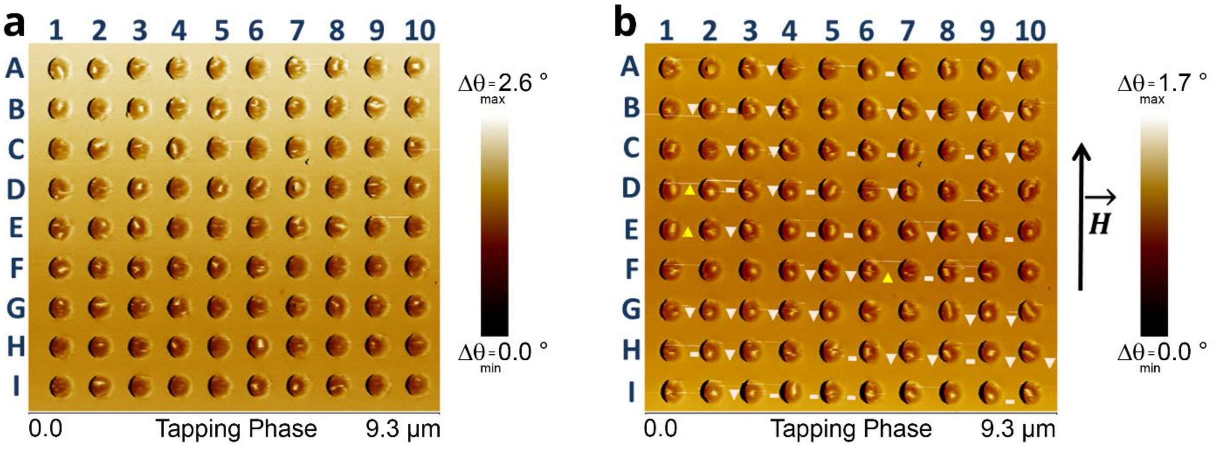

Figure S1 MFM images of a single array of 300 nm diameter disks sample type 15FP-AI. This figure covers the same sample of disks in Fig. 5 (5x7) but a larger array (9x10) for more statistics.

Figure S1a-b, shows a bigger array of disks (90 disks for better statistics) that went through the same field conditioning described in Fig. 5a-b (3 T out-of-plane field following an AC demagnetization process with a maximum 1 T in-plane field) including the same array of disks labeled with rows A-E in Fig. 5. In Fig. S1b, as compared to Fig. S1a, 36% of the dots moved downward whereas 3% moved upward and 22% of the dots appeared in the disk under in-plane external magnetic field of 10 mT. For the remaining 39% of the dots, the behavior is not clear.

MFM images of a set of disks under in-plane magnetic field

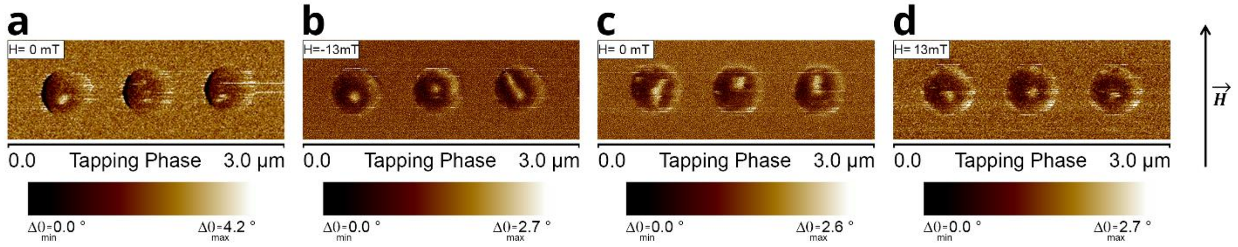

Figure S2 MFM images of a set of three 300 nm diameter disks from sample type 15FP-AI. The measurement was done after the field conditioning (-4 T out-of-plane field following an AC demagnetization process with a maximum 1 T in-plane field). MFM images taken under a) zero field, b) -13 mT, c) zero field after the application of -25 mT and d) 13 mT in-plane field.

The set of three disks with 300 nm diameter move anti-parallel to the in-plane magnetic field. MFM images were first taken under zero magnetic field and then -13 mT in-plane field. The radial vortices are observed to move upwards with the application of in-plane magnetic field in downward direction. -25 mT in-plane field was applied after the application of -13 mT, but the measurement was not possible due to effect of in-plane field to the magnetic tip. Hence, the disks are measured under zero field after the application of -25 mT in-plane field. The position of the radial vortices in this configuration can be explained considering the pinning effects. The radial vortices that went upwards further under -25 mT in-plane magnetic were pinned, enabling us to observe them under zero field. Later, the direction of the in-plane field was reversed, and the radial vortices were observed to move downward under 13mT in-plane field.

Determination of average domain width

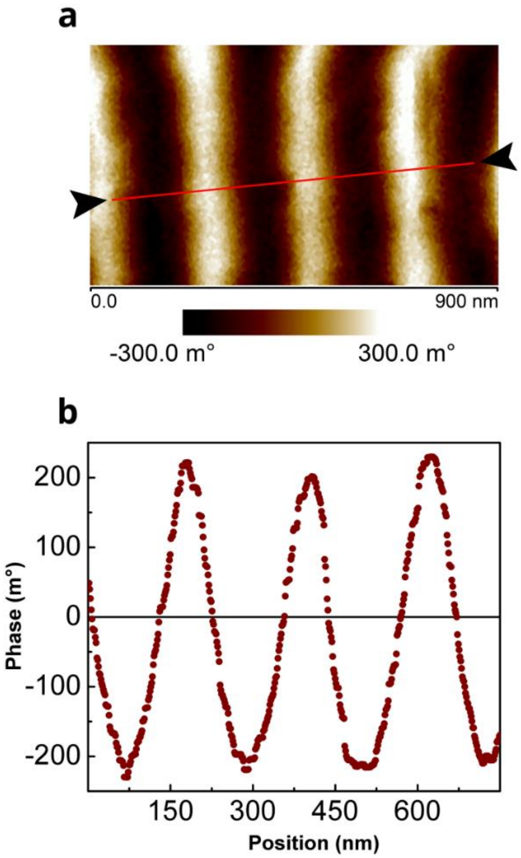

Figure S3 The section analysis for MFM a) MFM image shown to calculate the average domain width, b) Tapping phase (of magnetic tip) versus position graph obtained from the section line taken perpendicular to the stripe domains.

Magnetic Force Microscopy detects magnetic force gradient by measuring the phase difference of piezo drive and tip. The low moment tip with a nominal diameter of 50 nm senses the magnetic signal coming from the area underneath. To analyze the width of stripe domains seen in Fig. 3c and Fig. 3d, several cross-sectional lines were taken from the MFM images like the one shown in Fig. S2a. The average of maximum and minimum points (~0º) gives the approximate domain wall positions for the stripe domains which were used to calculate the average domain width.

Reconfirmation of Perpendicular Anisotropy in Sample 60C

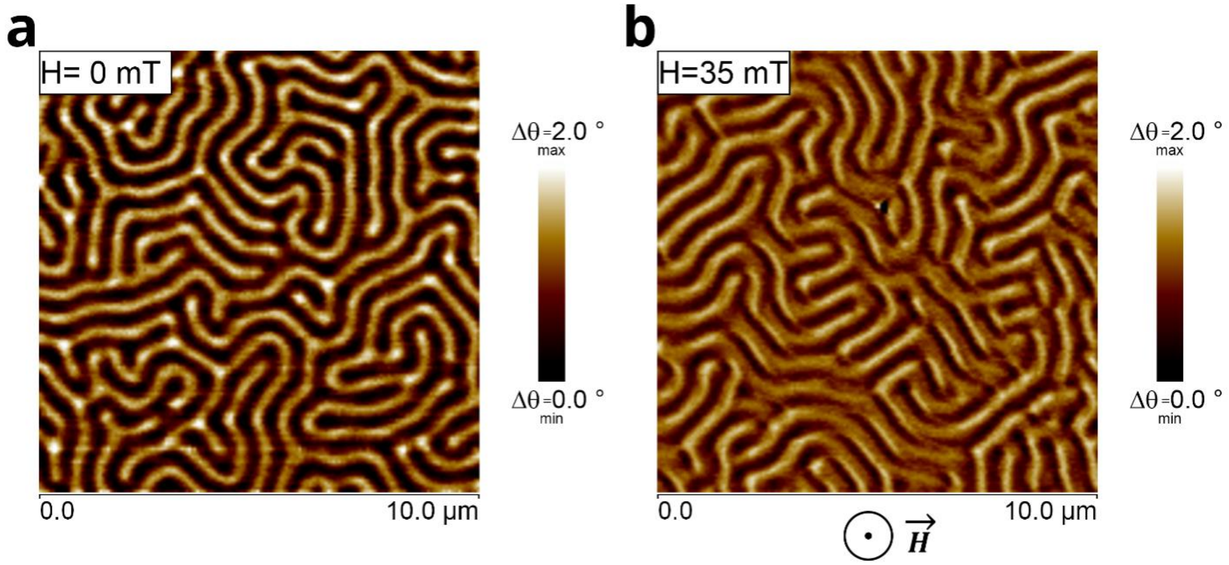

Figure S4 MFM images of sample 60C after in-plane AC demagnetization with a maximum field of 1 T a) with no external field b) under 35 mT external perpendicular to plane magnetic field in the indicated direction.

The VSM data for 60C with in-plane magnetic field shown in Fig. 3a suggests that the easy axis is out-of-plane. Similar to Fig. 3c for sample 15C-AI, we conducted a series of experiments under MFM to reconfirm PMA in sample 60C. The effect of increasing perpendicular to plane magnetic field from 0 to 35 mT was the growth of average size of the white domains from 265 nm to 330 nm, corresponding to 25% increase in average domain width.

Perpendicular to Plane External Field M-H curve of Samples 60C and 15C-AI

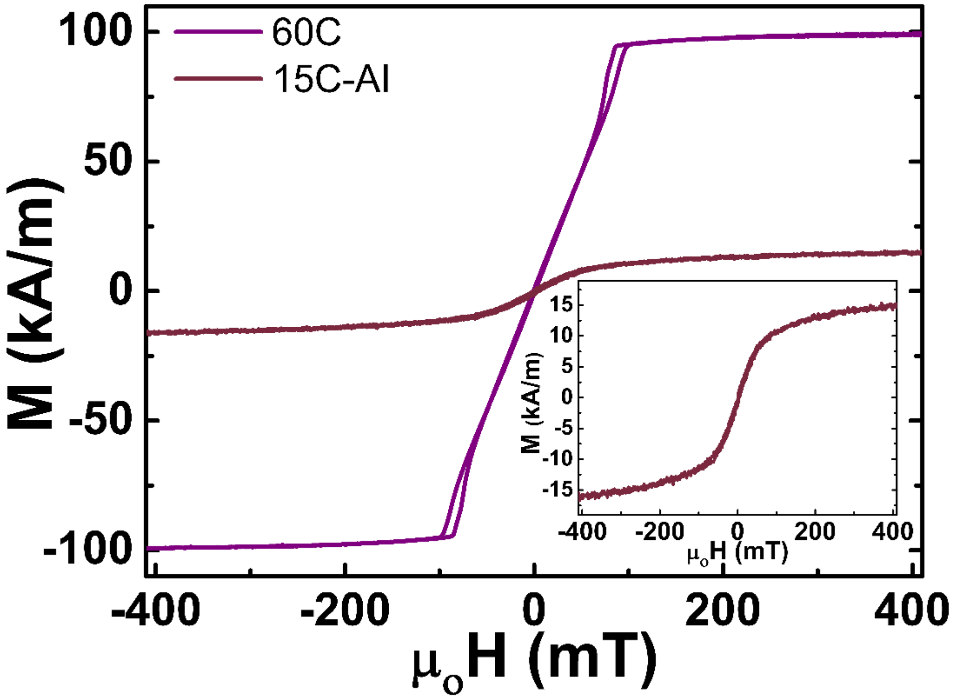

Figure S5 Room temperature VSM measurements with out-of-plane external magnetic field for samples 15C-AI (brown-red solid line) and 60C (purple solid line). The inset shows the zoomed in version for sample 15C-AI, showing the hard axis loop.

In order to check the in plane anisotropy in sample 15C-AI and perpendicular anisotropy of sample 60C out of plane M-H curves were also measured and are shown in Fig. S5.

Response of Néel Skyrmions and Radial Vortices to Perpendicular to Plane External Field

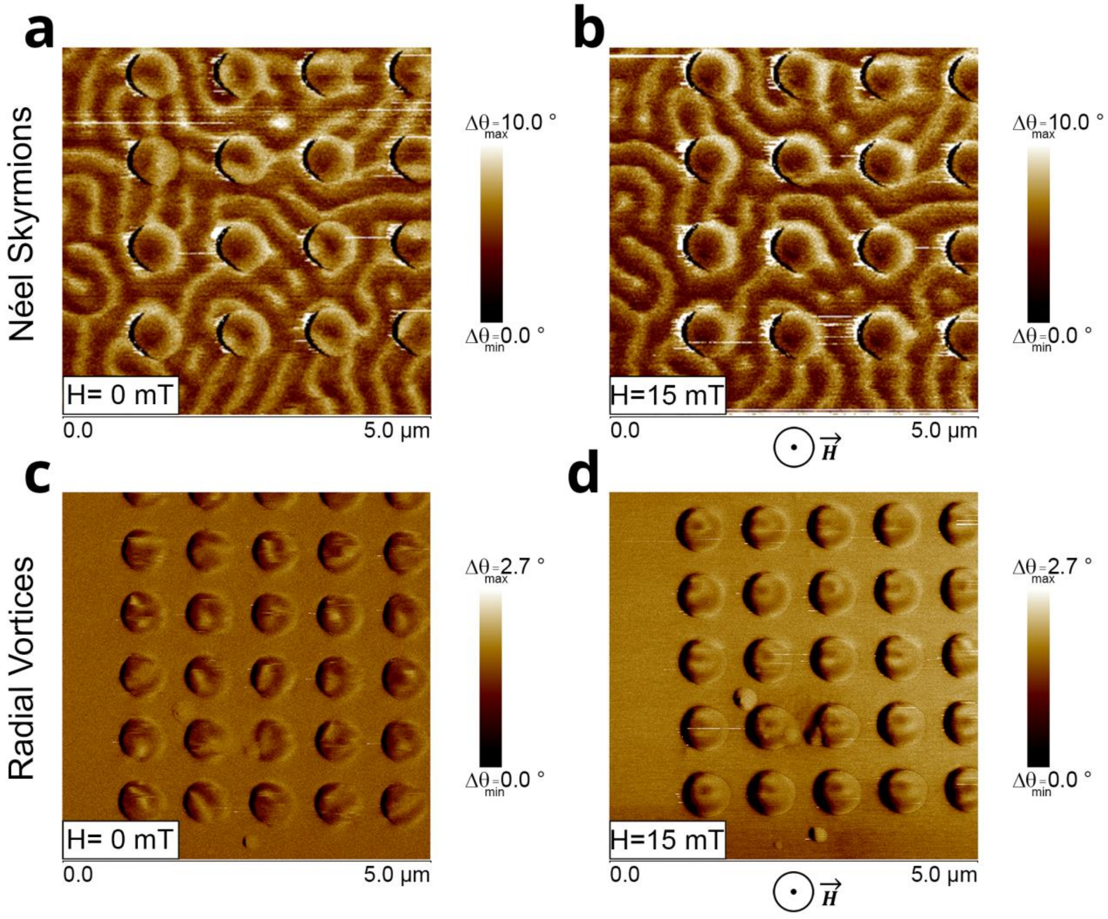

Figure S6 Response of Néel Skyrmions and Radial Vortices to Perpendicular to Plane External Field. MFM images of a-b) Néel Skyrmions on sample 15PP-A and c-d) Radial Vortices on sample 15FP-AI a-c) without an external field and b-d) under 15 mT out-of-plane magnetic field in the indicated direction.

Néel skyrmions in Fig. S4a with no external field expanded under out-of-plane magnetic field as shown in Fig. S4b. We conclude that our Néel Skyrmions demonstrate the expected behavior[1] as growing through the edges of the nano-disks. The extended radial vortices appearing under zero field in Fig. S4c respond to the same out-of-plane field of 15 mT in such a way that the

cores of the radial vortices expand somewhat and at the same time the pinning center induced extensions are eliminated.

Titanium Oxidation Analysis via X-Ray Photoelectron Spectroscopy

To further investigate the effect of substrate temperature on the oxidation of Ti interlayers and the accompanying change in lattice strain, we performed X-ray photoelectron spectroscopy (XPS) study on two different Ti thin film samples deposited at temperatures 20°C and 60°C. We cleaned a few top layers with in situ ion milling in the XPS chamber prior to the acquisition of XPS spectra in order to get rid of the surface contamination effects due to atmosphere contact.

Figure S5 shows an overlay of Ti 2p spectra for Ti films deposited at 20°C (Fig. S5a) and 60°C (Fig. S5b). Using CasaXPS software[2], the acquired spectra could only be fit by considering four oxidation states of titanium, namely Ti (0) (metallic), Ti(II) (TiO) and residual amounts of Ti(III) (Ti_2O_3) and Ti(IV) (TiO_2). There are two peaks for each state because of spin-orbit splitting effect in 2p orbitals ($2p_{3/2}$ and $2p_{1/2}$). Asymmetric Lorentzian and Gaussian/Lorentzian line shapes were used on Shirley background to account for the metallic Ti peak and oxide peaks, respectively. Constraints for peak locations (on binding energy axis), full width at half maxima (FWHM) and doublet splitting values were imposed according to the referenced values[3,4].

XPS results indicate that an increase in the temperature during sputter deposition from 20°C to 60°C promotes the oxidation of titanium, predominantly in the form of titanium monoxide. This also explains TiO peaks appearing in XRD spectra in Fig. 3b. From Table S1, 10% decrease in metallic Ti is accompanied by 7% increase in monoxide species and 3% increase in other oxide species.

	Ti – Metallic (atomic percent)	TiO (atomic percent)
Deposited at 20°C	59.97	34.51
Deposited at 60°C	49.22	41.77

Table S1: Percentage values of oxidation states of titanium in thin films deposited at 20°C and 60°C. Residual amounts of Ti(III) and Ti(IV) states are not listed here.

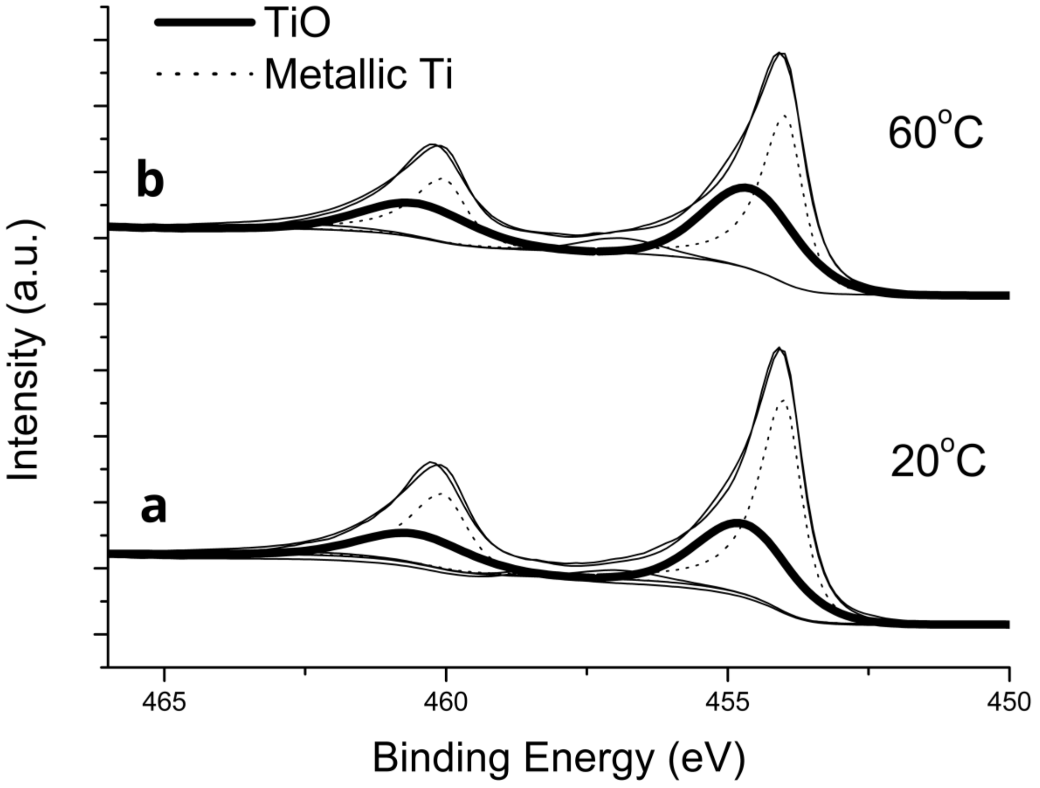

Figure S7 Overlaid XPS spectra of continuous Ti films deposited at a) 20°C and b) 60°C. Dotted and bold lines correspond to Ti 2p peaks of metallic titanium and titanium monoxide, respectively.

Micromagnetic simulations

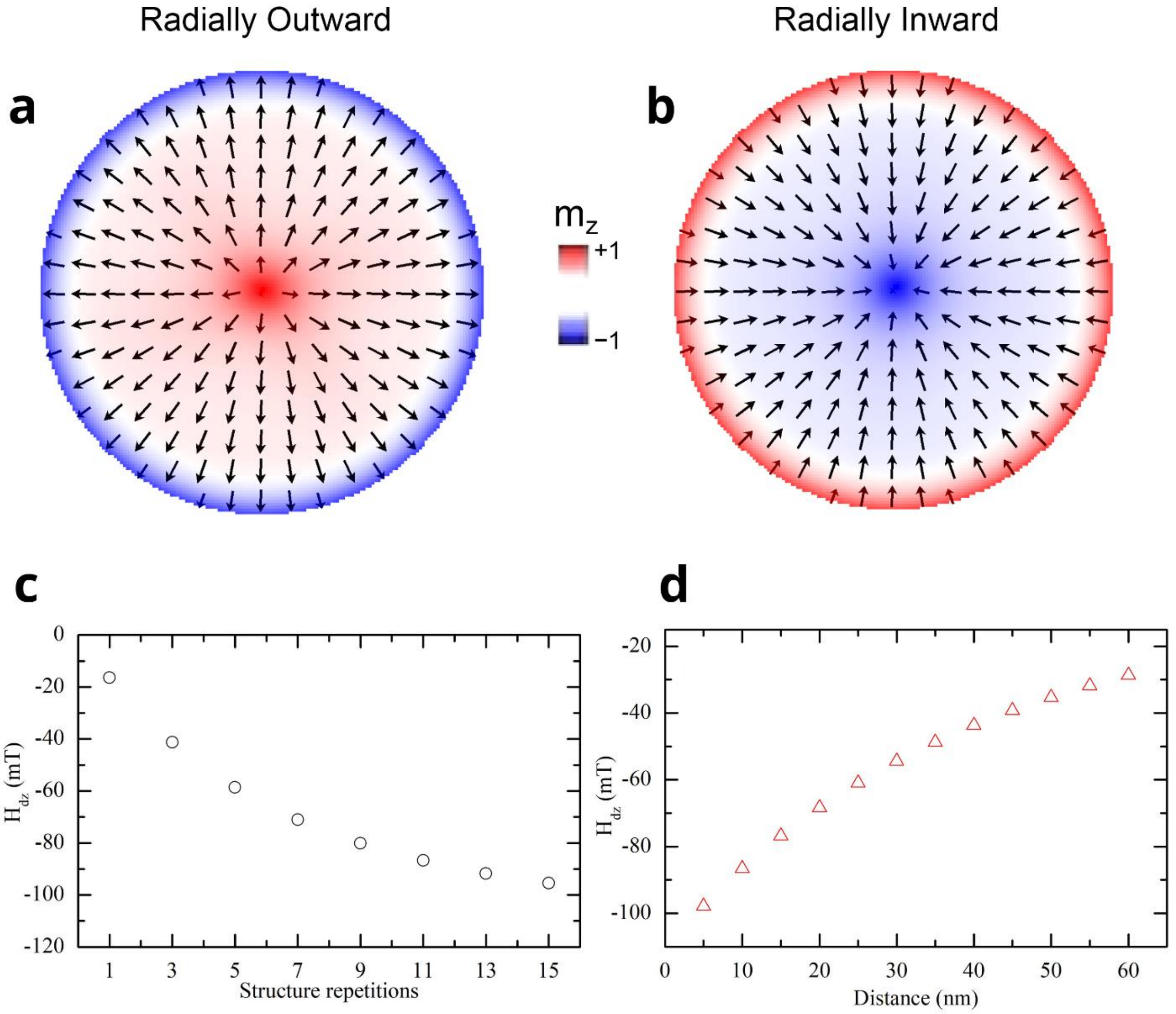

Figure S8 Micromagnetic simulation results obtained by using the experimental parameters indicate radially a) outward b) inward radial vortices. c) The dipolar field from the sample edge (left point) originated by an outward radial vortex as a function of number of repeats at a distance of 5nm from the surface d) The dipolar field from the sample as a function of tip distance from the surface for sample 15C-AI

The radial vortex spin configuration and the dipolar field from the samples as calculated by Micromagnetic simulations are shown in Fig. S8.

BLS Data for Samples 1C, 15C and 60C

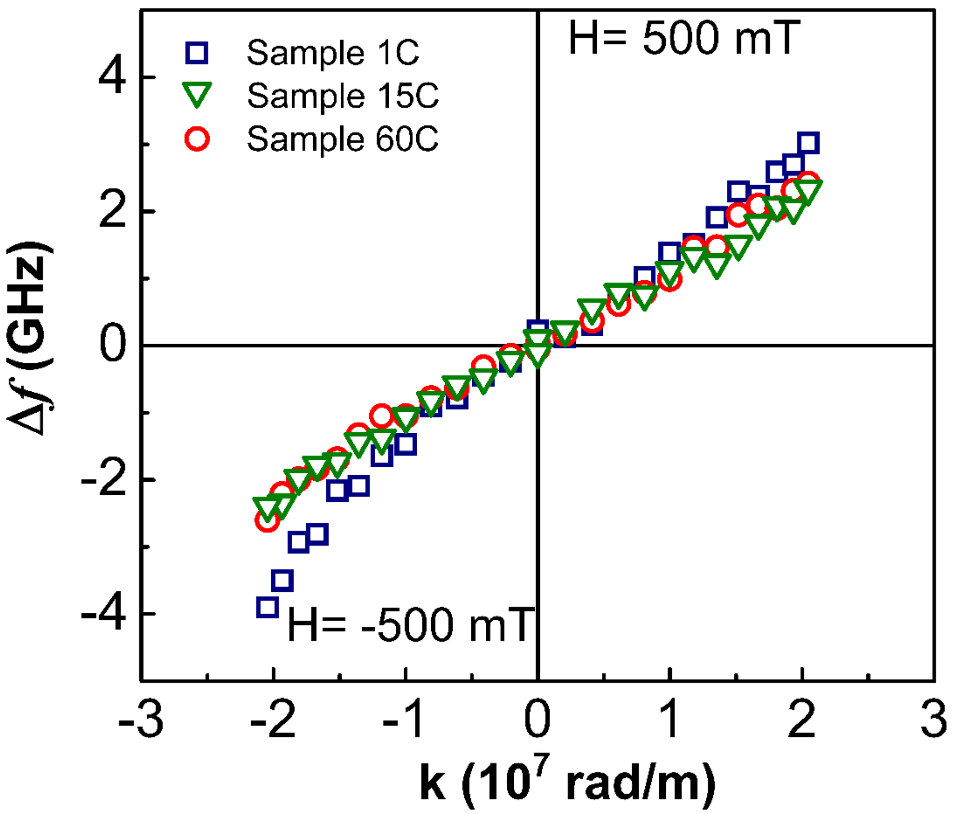

Fig S9. BLS Data for samples 1C, 15C and 60C

Supplementary References

1. Boulle, O. *et al.* Room-temperature chiral magnetic skyrmions in ultrathin magnetic nanostructures. *Nat. Nanotechnol.* **11,** 449–454 (2016).
2. Copyright © 2005 Casa Software Ltd. Available at: http://www.casaxps.com/. (Accessed: 26th July 2017)
3. A. V. Naumkin, A. Kraut-Vass, S. W. Gaarenstroom, and C. J. Powell, NIST Standard Reference Database 20, Version 4.1 (web version) (http://srdata.nist.gov/xps/), 2012.
4. Biesinger, M. C., Lau, L. W., Gerson, A. R. & Smart, R. S. Resolving surface chemical states in XPS analysis of first row transition metals, oxides and hydroxides: Sc, Ti, V, Cu and Zn. *Applied Surface Science* **257**, 887–898 (2010).
